# The Phagocytosis and Toxicity of Amorphous Silica

**DOI:** 10.1371/journal.pone.0014647

**Published:** 2011-02-02

**Authors:** Lindsey M. Costantini, Renée M. Gilberti, David A. Knecht

**Affiliations:** Department of Molecular and Cell Biology, University of Connecticut, Storrs, Connecticut, United States of America; University of Birmingham, United Kingdom

## Abstract

**Background:**

Inhalation of crystalline silica is known to cause an inflammatory reaction and chronic exposure leads to lung fibrosis and can progress into the disease, silicosis. Cultured macrophages bind crystalline silica particles, phagocytose them, and rapidly undergo apoptotic and necrotic death. The mechanism by which particles are bound and internalized and the reason particles are toxic is unclear. Amorphous silica has been considered to be a less toxic form, but this view is controversial. We compared the uptake and toxicity of amorphous silica to crystalline silica.

**Methodology/Principal Findings:**

Amorphous silica particles are phagocytosed by macrophage cells and a single internalized particle is capable of killing a cell. Fluorescent dextran is released from endo-lysosomes within two hours after silica treatment and Caspase-3 activation occurs within 4 hours. Interestingly, toxicity is specific to macrophage cell lines. Other cell types are resistant to silica particle toxicity even though they internalize the particles.

The large and uniform size of the spherical, amorphous silica particles allowed us to monitor them during the uptake process. In mCherry-actin transfected macrophages, actin rings began to form 1-3 minutes after silica binding and the actin coat disassembled rapidly following particle internalization. Pre-loading cells with fluorescent dextran allowed us to visualize the fusion of phagosomes with endosomes during internalization. These markers provided two new ways to visualize and quantify particle internalization. At 37°C the rate of amorphous silica internalization was very rapid regardless of particle coating. However, at room temperature, opsonized silica is internalized much faster than non-opsonized silica.

**Conclusions/Significance:**

Our results indicate that amorphous and crystalline silica are both phagocytosed and both toxic to mouse alveolar macrophage (MH-S) cells. The pathway leading to apoptosis appears to be similar in both cases. However, the result suggests a mechanistic difference between FcγRIIA receptor-mediated and non-opsonized silica particle phagocytosis.

## Introduction

Silicosis is one of the world's oldest known occupational diseases, characterized by irreversible, progressive lung disease due to the inhalation of crystalline silica. Although the disease is preventable with the proper precautions [1], many individuals are still at risk. The World Health Organization estimates over one million individuals in the United States are occupationally exposed to silica annually [2]. Silica (silicon dioxide) is one of the most abundant minerals on earth [3]; most commonly as sand, rock, and/or glass. Silica can be divided into two basic forms, crystalline and amorphous. Crystalline and amorphous silica have the same molecular formula [4], however the structural arrangements differ [5], crystalline silica lattices are regularly arranged while amorphous silica lattices lack order. Amorphous silica can be classified into three groups; (1) naturally occurring, (2) produced as a bi-product of power stations and metallurgical processing or (3) synthetically created [4], [5]. Synthetic amorphous silica is utilized in the manufacture of products such as pharmaceuticals, paints, cosmetics, and food as additives and fillers [6]. The increased use of synthetic amorphous silica could lead to higher instances of occupational amorphous silica exposure.

Crystalline silica is considered the toxic form of inhaled silica while less is known about the toxicity of amorphous silica. Epidemiological studies have drawn inconsistent conclusions on amorphous silica toxicity [7]. However, the underlying problem in evaluating the human effects of amorphous silica exposure is that there is usually some level of crystalline silica contaminating amorphous silica samples [5]. *In vivo* animal studies have reported a transient inflammatory response to amorphous silica [4], [8]. *In vitro* studies have shown that exposure of macrophages and macrophage cell lines to crystalline silica results in cell death by activation of the intrinsic apoptotic pathway [9], [10]. It is presumed that this cellular toxicity is an important aspect of the development of lung disease. However, much less is known about the *in vitro* toxicity of amorphous silica. We have recently demonstrated that crystalline silica is phagocytosed by macrophage cell lines [11]. We have now compared the uptake and toxicity of amorphous and crystalline silica.

## Materials and Methods

### Chemicals

Chemicals were purchased from Sigma (Sigma Chemical Co., St. Louis, MO) unless otherwise specified.

### Particles

Cells were treated with α-quartz crystalline silica, mean diameter 1.6 µm (Min-U-Sil 5; Pennsylvania Glass and Sand Corp., Pittsburg, PA), amorphous 3 µm spherical silica particles (Grace Davison Discovery Science formally Alltech, Deerfield, IL), amorphous 1 µm spherical silica particles (Spherotech Inc, Lake Forest, IL), or 3 µm polystrene latex beads (Polysciences, Inc., Warrington, PA),. Silica samples were washed three times with sterile distilled water and then baked overnight at 95°C to remove endotoxins. Particle concentrations for cell exposure were equalized for mass and plate area to 16.5, 25, or 50 µg/cm^2^. Particles were coated with ovalbumin (non-opsonized) or antibody (opsonized) as described previously [11].

### Cell culture

Mouse alveolar macrophages (MH-S, ATCC CRL-2019) cells were maintained in RPMI-1640 medium supplemented with 2 mM L-glutamine (Fisher Chemicals, Pittsburg, PA), 1 mM sodium pyruvate (BioWhittaker, Walkerville, MD), 10 mM HEPES (Fisher BioReagents, Pittsburg, PA), 10% fetal bovine serum (FBS) (Atlanta Biologicals, Lawrenceville, GA), 100 µg/ml ampicillin, and 100 µg/ml dihydrostreptomycin sulfate. RAW264.7 macrophages were maintained in DMEM medium containing 10% FBS. For experiments, cells were replated in plastic Falcon 30 mm, 6-well dishes, 24-well dishes (Becton Dickinson and Co. Fraklin Lakes, NJ), or Delta T glass-bottom culture dishes (Bioptechs, Inc, Butler, PA) at a density of 0.5–2×10^5^ cells/cm^2^ in RPMI-1640 complete medium and cultured overnight at 37°C and 5% CO_2_. Thirty minutes prior to the start of an experiment, the medium was changed to CO_2_-independent medium without L-glutamine (Invitrogen Corp., Carlsbad, CA) and the cells were maintained in a 37°C ambient air incubator until use.

Mouse lung epithelial type II (MLE ATCC #CRL-2110) cells were maintained in HITES medium, a 50∶50 mix of Dulbecco's medium (DMEM) and Ham's F12 supplemented with insulin (0.005 mg/ml), transferrin (0.01 mg/ml), 30 nM sodium selenite, 10 nM hydrocortisone, 10 nM β-estradiol, 10 mM HEPES, 2 mM L-glutamine, and 2% FBS. Thirty minutes prior to experiments, the medium was changed to HITES sodium bicarbonate-free medium buffered with 10 mM HEPES. Mouse skin melanoma (B16-F1, ATCC #CRL-6323), monkey kidney fibroblast (COS-7, ATCC #CRL-1651), and human adenocarcinoma cervical epithelial (HeLa ATCC #CCL-2) cells were cultured in Dulbecco's Modified Eagle's medium (DMEM) supplemented with 2 mM L-glutamine, glucose (3.5 mg/ml) (Fisher Chemicals, Pittsburg, PA), 10% FBS, 100 µg/ml ampicillin, and 100 µg/ml dihydrostreptomycin sulfate. Madin-Darby canine kidney epithelial (MDCK, ATCC CCL-34) and rat mammary adenocarcinoma (MtLN3, [12]) cells were cultured in Minimal Eagle's medium (MEM) supplemented with 2 mM L-glutamine, 2 mM NEAA (HyClone, Logan, UT), 10% FBS, 100 µg/ml ampicillin, and 100 µg/ml dihydrostreptomycin sulfate. Mouse embryo fibroblast (NIH-3T3 ATCC #CRL-1658) cells were maintained in DMEM supplemented with 2 mM L-glutamine, glucose (3.5 mg/ml) (Fisher Chemicals, Pittsburg, PA), 10% fetal calf serum, 100 µg/ml ampicillin, and 100 µg/ml dihydrostreptomycin sulfate. Thirty minutes prior to experiments, medium for B16-F1, COS-7, HeLa, MDCK, MtLN3, and NIH3T3 cells was changed to HAM's F12 media buffered with 10 mM HEPES and supplemented with 10% FBS.

### Microscopy

DIC images, or paired DIC and fluorescence images, were collected sequentially using a Zeiss Axiovert 200 M microscope (Zeiss, Göttingen, Germany) with a 63× oil objective or 40× dry objective. Images were acquired using a Hamamatsu ORCA-ER camera (Hamamatsu, Bridgewater, NJ). OpenLab software (Improvision, Inc., Portage, MI) was used for image acquisition and hardware control. Time-lapse images were then converted to Quicktime movies (Apple, Inc., Cupertino, CA).

### Cell death assay

To measure the amount of cell death induced by various particle types, cells were cultured in Bioptechs glass bottom dishes or 6-well tissue culture plates. Media was replaced with CO_2_-independent Media (Invitrogen, Inc.) and cells were incubated at 37°C, with atmospheric CO_2_ in the presence of 0.1 µg/mL propidium iodide (PI). Cells were treated with no particles, 3 µm latex beads, crystalline silica, or 3 µm amorphous silica at varying concentrations for 24 hours. Cell death was measured by counting the proportion of cells with PI staining of the nucleus at 0, 4, 8, 12 and 24 hour timepoints.

### Western Blotting

Western blotting was performed as described previously [9], [10]. Briefly, MH-S cells were treated with no particles, latex beads, crystalline, or spherical silica [25 µg/cm^2^] for 4 or 8 hours. Floating and adherent cells were harvested in the medium and centifuged at 4,000 rpm for 5 minutes, after which the supernatant was removed. The lysis buffer (RIPA lysis buffer: PBS with 1% Nonidet P-40, 0.5% sodium deoxycholate, 0.1% SDS containing freshly added protease inhibitors: 2 mM phenylmethanesulfonly fluoide (PMSF), 10 µg/ml apoprotinin, 10 µg/ml pepstatin, and 10 µg/ml leupeptin hemisulfate) was added to the cell pellet incubated on ice for one hour. Samples were then centrifuged at 10,000X g for 10 minutes at 4°C, and the supernatants were removed and stored at -20°C. Samples were resolved on a 12.5% SDS-polyacrylamide gel and electrotransferred to nitrocellulose. Blocking was done with TBS/0.1% Tween20/5% w/v low fat milk for one hour at room temperature. Immunodetection for Caspase-3 was done with rabbit anti-Caspase-3 (Cell Signaling Technology Inc., Beverly, MA) followed by alkaline-phosphatase goat anti-rabbit antibody. Bands were visualized colorimetrically (p-nitroblue tetrazolium chloride (NBT), 5-Bromo-4-chloro-3-indolylphosphate disodium (BCIP) (Research Organics Inc., Cleveland, OH).

### Quantitation of phagocytosis by antibody staining

Particle internalization was quantified by a modification of the previously described assay [11]. Cells were plated at 1×10^5^ cells/cm^2^ on 25 mm glass cover slips (Fisher Scientific, Pittsburg, PA) in 30 mm Falcon dishes in RPMI-1640 complete medium and allowed to adhere overnight. The medium was then replaced with CO_2_-independent medium at 37°C in an ambient CO_2_ incubator. Opsonized (ovalbumin plus rabbit anti-ovalbumin-coated) or ovalbumin-coated 3 µm spherical silica [16.5–25 µg/cm^2^] was added to cells. The zero time sample was immediately fixed by adding an equal volume of medium containing 8% formaldehyde (Polysciences, Inc.) and the remaining plates were placed in the 37°C atmospheric incubator and then fixed at the indicated times. The fixation solution was removed after 6 minutes and replaced gently with 50 mM NH_4_Cl and incubated for 5 minutes at 25°C and then washed with PBS at pH 7.4, for one minute at 25°C. PBS was gently aspirated and replaced with 200 µl of 1∶800 rabbit anti-ovalbumin antibody (Immunology Consultants Laboratory, Newberg, OR). After 35 minutes at 25°C, the primary antibody was removed; the coverslips washed with PBS and then incubated with 200 µl of 1∶150 FITC-conjugated goat anti-rabbit antibody (Jackson ImmunoResearch, West Grove, PA) for 35 minutes at 25°C. The secondary antibody was removed and PBS was used to wash the coverslips twice, followed by one wash with distilled water. The coverslips were mounted on glass microscope slides using 10 µl of 10% DABCO and 2.5% MOWIOL mounting media. The antibodies cannot penetrate the membrane of the fixed cells, so only external particles fluoresce. To determine particle internalization, total particles and internalized particles were determined visually and the data is presented as percent of cell associated particles that are internalized.

### Measurement of phagocytosis at different temperatures

Cells were plated at 1×10^5^ cells/cm^2^ on 25 mm round glass coverslips in 30 mm dishes in RPMI-1640 complete media and allowed to adhere overnight. Media was replaced with 500 µl of CO2- independent media for 15 minutes in an atmospheric 37°C incubator. Ovalbumin-coated or opsonized particles were then added to cells (final concentrations: 15 µg/cm2 of silica, or 40 µl of 2.5% latex beads) and the plates were centrifuged at 300×g for 5 minutes at 21°C. One set of coverslips was immediately fixed and stained as indicated above. To rapidly increase the temperature of the 37°C sample, 500 µl of 37°C medium was added and the plates were placed in a 37°C atmospheric gas incubator. The other dishes received room temperature medium and were incubated at room temperature (approximately 22°C).

### Actin localization during phagocytosis

To investigate the involvement of F-actin at the site of particle internalization, MH-S cells were transfected with mCherry-actin using electroporation (manuscript in preparation). Stable cell lines were isolated by selection in G418 for 2 weeks. Clonal colonies with a high proportion of fluorescent protein expression were identified visually and then picked manually with a pipettman. Expressing cells were plated in a Bioptechs dishes and mounted on the Delta T stage incubator set at 37°C. Cells were treated with no particles, 3 µm latex beads, crystalline silica, or 3 µm amorphous silica at varying concentrations. Fluorescence images were captured every 30 seconds for up to 45 minutes using a Zeiss 200 M inverted microscope.

In order to quantify particle internalization in live cells, the appearance and disapperance of actin fluorescence around the particle was used. A ring of fluorescent actin associated with F-actin polymerization appears around particles as they began the uptake process. After internalization, the particle can be seen as a black circular hole in the background of fluorescent cytoplasmic G-actin. DIC and fluorescence image sequences were evaluated to determine the proportion of particles internalized over time.

### Endosome-Phagosome Fusion and Endo-lysosomal Leakage

To evaluate endo-lysosomal leakage caused by silica, cells were first pre-loaded with 20 kD Fluorescein isothiocyanate (FITC)-conjugated dextran (Sigma, St. Louis, MO) which localized to endo-lysosomal vesicles via endocytosis [10]. Following overnight plating in Bioptech dishes, the medium was removed and replaced with RPMI-1640 complete medium containing 1 mg/mL FITC-dextran. The cells were incubated for 90 minutes at 37°C, 5% CO_2_ and then washed three times with RMPI-1640 and incubated in 1 ml of fresh RPMI-1640 complete medium for 5 minutes at 37°C. The cells were then washed three additional times with RMPI-1640 and finally with CO_2_-independent medium. Cells were then treated with or without silica and imaged. Dual DIC and fluorscence images were taken every 5–15 minutes for 3–6 hours using OpenLab automation for timelapse.

To quantify particle uptake using dextran fluorescence, cells were labeled as indicated above and then exposed to 3 micron amorphous silica particles. Time lapse images were acquired every 30 seconds in both the FITC and DIC channels. Because the phagosome is higher in pH than endosomes, the particles become brightly labeled shortly after uptake upon endosome-phagosome fusion. The number of cell-associated non-fluorescent and fluorescent particles was determined visually at each time point in order to quantify the kinetics of uptake.

### Statistical analysis

Experiments were replicated at least three independent times, on different days. Error bars represent standard error. P-values were calculated using ANOVA testing.

## Results

### Both crystalline and amorphous silica induce cell death in macrophages

**Figure 1:**
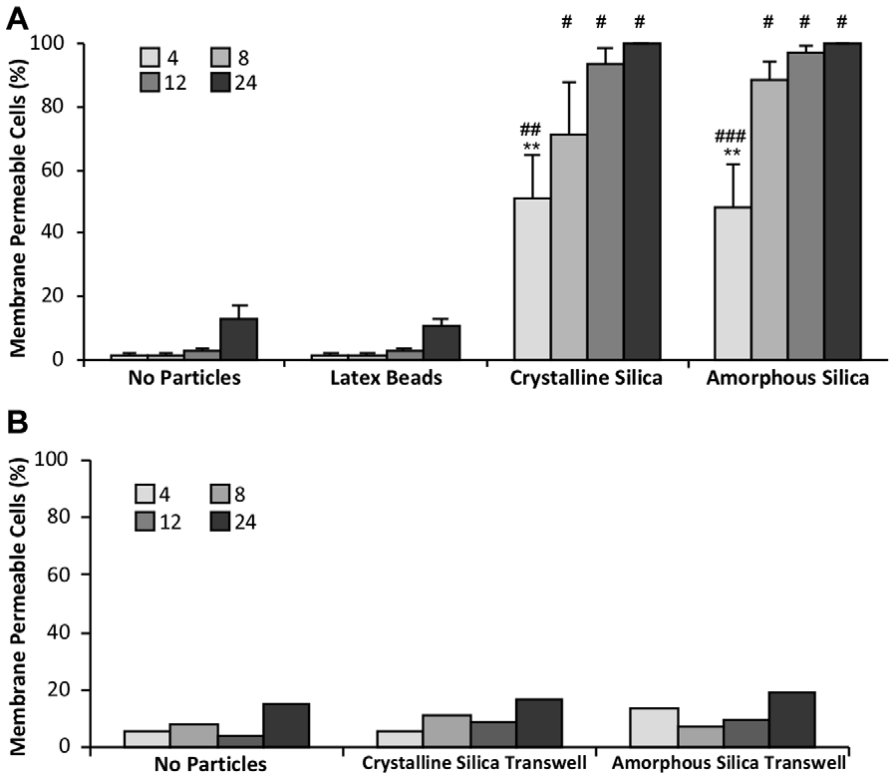
Both crystalline and amorphous silica induce high rates of cell death in macrophages. MH-S cells treated with no particles, 3 µm latex beads, crystalline silica [50 µg/cm^2^] or 3 µm spherical, amorphous silica [50 µg/cm^2^]. (**A**) Cell death measured by propidium iodide (PI) [0.1 µg/ml] nuclear staining at 4, 8, 12 and 24 hours. Statistically significant differences compared using ANOVA, (* = P<0.001, ** = P<0.01, as compared to untreated control, # = P<0.001, ## = P<0.01, ### = P<0.05, as compared to latex beads). (**B**) Crystalline or amorphous silica [50 µg/cm^2^] was placed in a Transwell (0.45 µm diameter pore size) chamber and placed above cells attached to the bottom of a 24 well culture dish. Cell death was measured with PI (n = 150–600 cells).

It is well known that exposure of mouse alveolar macrophage (MH-S) cells to crystalline silica induces cell death [9], [13]. In order to assess the toxicity of amorphous silica, cells were exposed to spherical, amorphous particles. Both crystalline and amorphous silica induced high amounts of cell death in MH-S cells (Fig. 1A). Four hours after addition of high amounts of silica (50 µg/cm^2^), 51% of cells exposed to crystalline silica and 48% of cells exposed to amorphous silica had propidium iodide (PI) stained nuclei. Twenty-four hours after silica treatment, 100% of cells were killed by both silica types (Fig. 1A). These results demonstrate that both crystalline and amorphous silica are toxic to MH-S cells. Similar results were found using RAW264.7 macrophage cells (Fig. S1). We also examined additional types of silica particles. One micron amorphous silica particles also induced cell death at comparable rates to 3 µm amorphous silica (data not shown). Amorphous silica particles from different manufacturers had similar effects on mouse alveolar macrophage cell lines.

To verify that cell death was due to the direct interaction of MH-S cells with silica and not soluble factors contaminating the particle surface or produced by silica-media interaction, cells were cultured in the presence of the particles but prevented from making direct contact with them. Cells were plated in 24-well culture dishes and then Transwell inserts with a 0.45 µm diameter pore size filled with either crystalline or amorphous silica [50 µg/cm^2^] were inserted into the wells containing the cells. This allowed media exposed to particles to interact with cells below the Transwell membrane barrier. There was no increase in toxicity caused by the presence of particles in the Transwell chambers (Fig. 1B). These results indicate that cell death is caused by the direct interaction of the cells with both types of particles.

### MH-S mCherry-actin cells internalize amorphous silica and latex beads at equal rates

**Figure 2:**
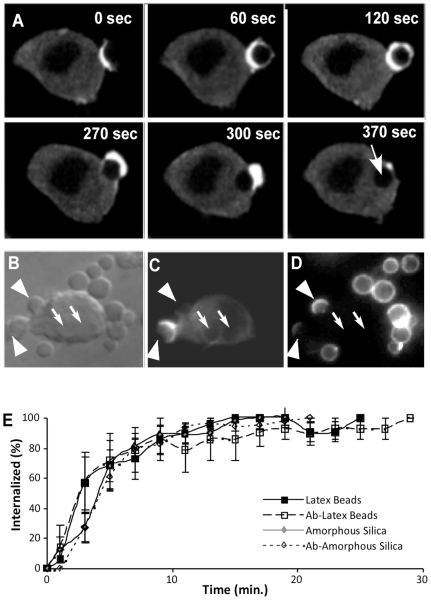
Actin localization at the site of amorphous silica particle phagocytosis. (**A**) MH-S mCherry-actin transfected cells were exposed to 3 µm spherical, amorphous silica and images were captured every 30 seconds. The accumulation of fluorescence, indicating the polymerization of F-actin filaments, can be seen at the point of cell-particle contact. The actin ring extends around the particle and the filaments depolymerizes as the particle is internalized. The arrow in the 370 second panel shows the location of the particle which can be seen as an unlabeled black hole in the G-actin background fluorescence of the cytoplasm. (**B–D**) MH-S mCherry-actin transfected cells were treated with ovalbumin-coated 3 µm spherical, amorphous silica then fixed and stained with rabbit anti-ovalbumin and then goat anti-rabbit FITC-conjugated antibody to determine if particles were inside or outside the cell membrane. (**B**) DIC image (**C**) mCherry actin image (**D**) FITC-antibody labeled external particles. Arrows indicate the location of internal particles. Arrowheads indicate the location of external particles. Scale bar = 5 µm. (**E**) The rate of uptake as measured by examining the appearance of actin rings and subsequent appearance of a particle sized black hole in the cytoplasm.

We have recently shown that non-opsonized silica particles and latex beads are internalized by macrophages [11]. The mechanism of internalization of non-opsonized particles is unknown. In order to determine whether actin filament polymerization plays a role in internalization, particle uptake in MH-S cells expressing mCherry-actin was imaged after exposure to silica particles. The sequence of images in Figure 2A shows the changes in mCherry-actin localization during the uptake of a non-opsonized 3 µm amorphous silica particle. Initially, as the cell binds the particle, F-actin begins to accumulate at the site of particle contact, forming an actin rich phagosomal cup. As phagocytosis proceeds, the pseudopods fuse on the distal side of the silica particle forming a complete actin ring (120 seconds; Fig. 2A). By 270 seconds, the particle is partially internalized and the actin filaments on the proximal side of the cup have already depolymerized. As the particle is fully internalized, the actin filaments on the distal side of the particle also disappear and the internalized particle can still be seen in the fluorescence images as a black hole in the cytoplasm (Fig. 2A, 390 seconds). Although the particles used in this experiment were non-opsonized, and thus IgG antibody was not present, the process of uptake is nonetheless very similar to that seen during FcgammaRIIA receptor-mediated uptake of antibody-coated particles[14], [15]. Actin accumulation was also seen at the sites of phagocytosis of irregularly-shaped crystalline silica and 1 µm spherical silica (data not shown). However, due to the small size and/or irregular shape of these silica particles, visualization of uptake was more difficult.

To confirm that the actin ring signified phagosome formation and that the round black hole in the cytoplasm indicated an internalized particle, MH-S mCherry-actin cells were incubated with ovalbumin-coated (non-opsonized) 3 µm amorphous silica particles and after 5 minutes, fixed and stained with rabbit anti-ovalbumin and goat anti-rabbit FITC antibodies. Since fixed membranes are not permeable to antibody, only external particles were fluorescently labeled. In the DIC image (Fig. 2B), the arrows show the location of internal particles. These particles appear as round black holes in a background of cytoplasmic fluorescence, indicating internalized particles in which the phagosomes have lost the actin coat. The cytoplasmic localization is confirmed by the absence of fluorescent staining by anti-ovalbumin antibody (Fig. 2D). The image of the mCherry-actin fluorescence shows accumulation of actin filaments at the site of the particle undergoing phagocytosis (Fig. 2C, arrowhead). External particles (Fig. 2D, arrowheads) are fluorescently stained by the antibody, while internalized particles are unstained (Fig. 2D, arrows). The particle that is undergoing phagocytosis is partially stained by the antibody, indicating that it is not yet fully engulfed by the membrane (Fig. 2D). This result suggests that the particle-cell association is a tight interaction that excludes the antibody from the site of phagocytosis.

Actin accumulation at the site of particle phagocytosis was utilized as a method to quantify the rate of particle internalization in expressing cells (Fig. 2E). mCherry-actin expressing cells were exposed to opsonized or non-opsonized spherical, amorphous silica or latex beads and time lapse images of fluorescence and DIC were collected. The image sequence was examined to assess when actin accumulation occurred and when a cytoplasmic black hole formed as a measure of uptake. There was no significant difference in the uptake of any of the particle types and within fifteen minutes, nearly 100% of all particles were internalized.

### Temperature affects the rate of particle internalization

**Figure 3:**
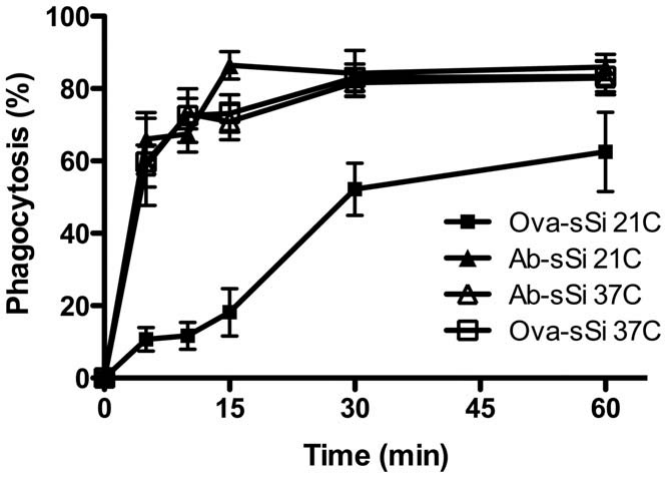
Temperature affects opsonized and non-opsonized particle phagocytosis differently. MH-S macrophages were exposed to ovalbumin coated (Ova) or ovalbumin plus anti-ovalbumin antibody coated (Ab) 3 µm spherical, amorphous silica. The cells were either maintained at 37°C throughout the assay or incubated at room temperature (RT, about 22°C,) during particle exposure. Ova-RT: closed squares; Ova−37°C: open squares; Ab-RT: closed triangles; Ab−37°C: open triangles. Cells were fixed at the indicated times and particle internalization was measured by staining with anti-ovalbumin antibody.

Using a different protocol, we had previously found that internalization of non-opsonized silica was significantly slower than the uptake of opsonized particles [11]. To resolve this discrepancy, differences between the antibody staining assay and the actin accumulation assay were examined. First, we used the actin accumulation assay to compare the uptake of uncoated particles with particles coated with either ovalbumin (non-opsonized) or ovalbumin followed by anti-ovalbumin antibody (opsonized) at 37°C. Coating had no effect on either the rate or the extent of internalization of particles (data not shown). One of the key differences between the assays is that the actin assay is done at 37°C on a heated microscope stage whereas the antibody staining assay was done in a 37°C incubator. However, in order to synchronize particle delivery in the latter assay, cells were chilled on ice for ten minutes prior to silica exposure and then moved to the incubator. This results in a gradual warming of the cells to 37°C. To test whether temperature differentially affected uptake, phagocytosis of the particles was measured at two different temperatures using the actin ring assay (data not shown) and antibody-staining assay. Both opsonized and non-opsonized amorphous silica particles were rapidly internalized at 37°C. At room temperature, however, the opsonized particles were rapidly internalized, but the non-opsonized particles were taken up significantly more slowly (Fig. 3). Similar results were obtained with latex particles (data not shown) and with RAW264.7 macrophage cells (Fig. S2). This result indicates that there is a temperature-sensitive step in the non-opsonized particle uptake pathway and reflects a mechanistic difference in the two pathways.

### Crystalline and amorphous silica can induce endo-lysosomal leakage in MH-S cells

**Figure 4:**
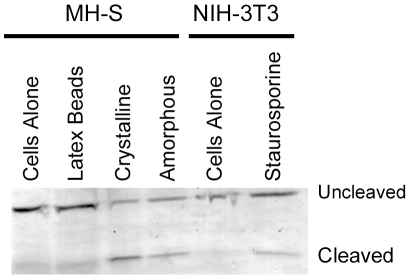
Activation of Caspase-3 in amorphous silica treated cells. Western Blot of MH-S cells treated with latex beads, crystalline silica [50 µg/cm^2^], or 3 µm amorphous silica [50 µg/cm^2^] for 4 hours. Blots were probed with anti-Caspase-3 antibodies and visualized colorimetrically. Cleavage of the 35 kD Caspase-3 to the 19 kD active form could be detected in both amorphous and crystalline silica treated cells but not cells treated with latex beads. As a positive control, NIH-3T3 cells were treated with staurosporine for 1 hour.

In order to determine whether the mechanism of induction of cell death by amorphous silica was similar to that found for crystalline silica, several key aspects of the cell death pathway were examined. It has previously been shown that crystalline silica induces endo-lysosomal leakage and cleavage of the apoptotic marker Caspase-3 [10]. Cells were treated with silica and then the activation of Caspase-3 was determined by Western blotting. The ratio of cleaved (active) to uncleaved (inactive) Caspase-3 increased dramatically following treatment with either crystalline or amorphous silica (Table 1 and Figure 4) indicating activation of the apoptotic pathway.

**Table 1:** Quantification of Caspase-3 cleavage.

Treatment	Cell Type	Cleaved/Uncleaved Caspase-3 Ratio
Cells Alone	MH-S	0.23
Latex Beads	MH-S	0.31
Crystalline	MH-S	1.35
Amorphous	MH-S	0.67
Cells Alone	NIH-3T3	0.15
Staurosporine	NIH-3T3	0.27

The bands associated with uncleaved and cleaved Caspase-3 in Western blots were quantified and the ratio determined for control and silica treated cells. Staurosporine treated NIH-3T3 cells was used as a positive control for apoptosis.

**Figure 5:**
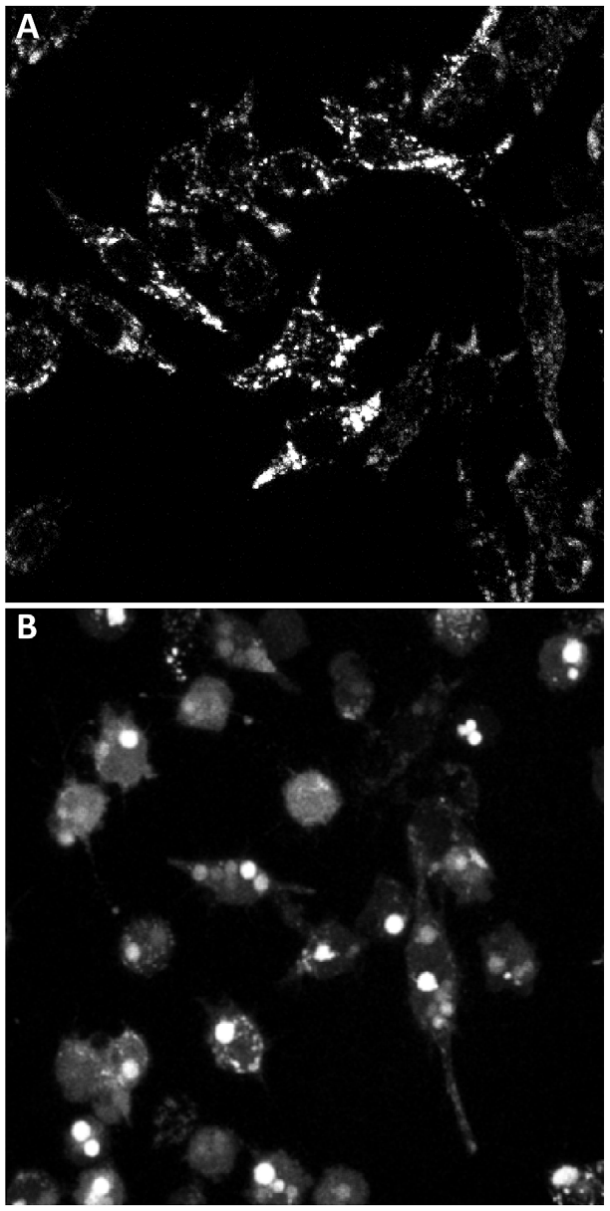
Amorphous silica induces endo-lysosomal leakage in MH-S cells. MH-S cells are pre-loaded with 20 kD FITC-dextran [1 mg/ml] for × hours. Fluorescence images of cells untreated cells (**A**) or treated with 3 µm spherical, amorphous silica particles [25 µg/cm^2^] for 1.5 hours (**B**). The punctate endosomal distribution of FITC-dextran is converted to a diffuse cytoplasmic localization after treatment of cells with amorphous silica.

To test whether amorphous silica induces endo-lysosomal leakage, cells were pre-loaded with FITC-dextran and then exposed to either 3 µm or 1 µm spherical, amorphous silica. Prior to silica exposure, the endo-lysosomal vesicles have a punctate pattern that is faint due to quenching of the FITC probe in the acidic environment of the endosomes and lysosomes (Fig. 5A). As early as one hour after silica addition, the punctate patterning is lost and a bright diffuse cytoplasmic signal is observed, indicative of endo-lysosomal leakage (Fig. 5B and Movie S1). These results are consistent with the endo-lysosomal leakage seen previously with crystalline silica treated macrophages [10].

### FITC-dextran particle labeling is an additional method to quantify the rate of particle internalization

**Figure 6:**
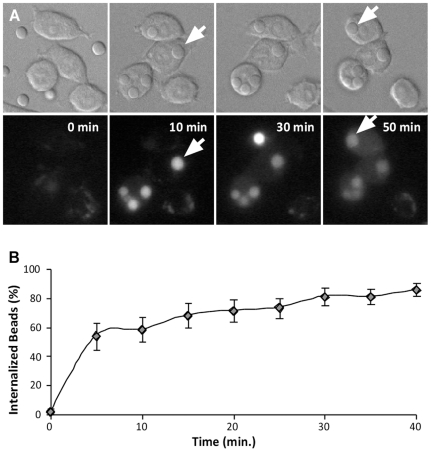
FITC-dextran particle labeling can be used to quantify the rate of particle internalization. MH-S cells are pre-loaded with 20 kDa FITC-dextran [1 mg/ml]. (**A**) Paired DIC and fluorescent images showing FITC-dextran particle labeling during uptake. Arrows indicate internalized particles, scale bar = 5 µm. (**B**) The rate of 3 µm uncoated amorphous silica [16.5 µg/cm^2^] internalization using this assay (n = 26 cells).

We noticed that as amorphous silica particles were internalized by cells pre-loaded with FITC-dextran, the particles in the phagosomes became fluorescently labeled during uptake (Fig. 6A, arrows). This is presumably due to the fusion of endosomes containing fluorescent dextran with the nascent phagosome. Because the silica particles are porous, they are labeled throughout their volume, unlike latex beads. Amorphous silica particles initially stained very bright, and then the signal decreased to a steady state until the leakage occurred. Because the FITC dye is pH sensitive, the decrease in brightness of the phagosome most likely reflects the acidification of the phagosome by proton pumps [16]. This observation allowed us to develop an additional uptake assay for live cells based upon particle labeling by fluorescent dextran. Using this assay, rapid uptake can be seen within five minutes of silica exposure (53.8±9.16%, n = 26 cells) and reaches 68.0±8.4% internalization by fifteen minutes after silica addition (Fig. 6B). These results are consistent with the previous rates of internalization measured by the fixed cell or the actin accumulation phagocytosis assays. The results demonstrate that these phagosomes are processed similarly to opsonized particle phagosomes and that FITC-dextran particle labeling can be used as an additional method to quantify the rate of particle internalization.

### One internalized particle is sufficient to induce cell death in MH-S cells

**Figure 7:**
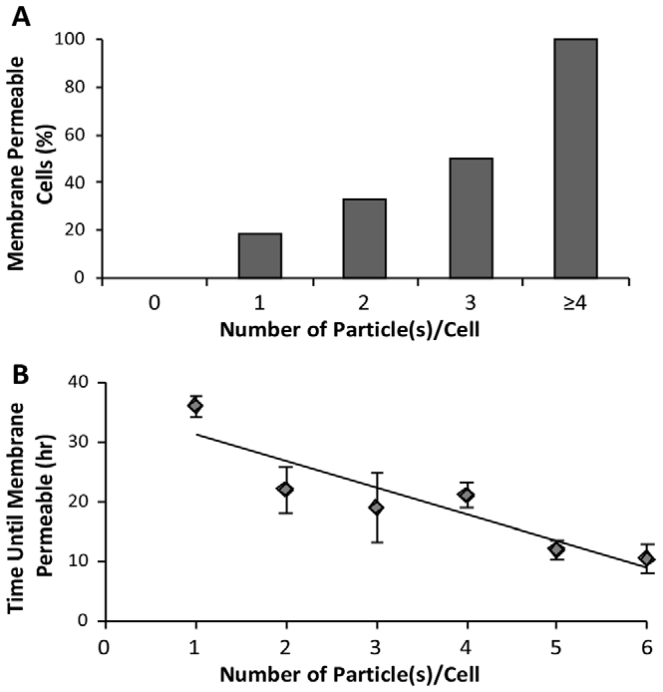
The number of internalized particles affects the timing of cell death. MH-S mCherry-actin cells were treated with amorphous silica [16.5 µg/cm^2^-25 µg/cm^2^] and the time and number of particles internalized was determined for each cell. Time lapse imaging was continued in the presence of PI to monitor if and when cell death occurred. (**A**) The number of particles (per cell) that induces cell death as measure by the percent of PI positive cell nuclei, (n = 38 cells at 16 h). (**B**) Average length of time before a cell with a specific number of particles dies, (n = 37 cells).

The number of silica particles required to cause cell death is unknown. With the availability of a live cell uptake assay, it became possible to directly address this question. Spherical 3 µm amorphous silica particles were added at low concentrations to MH-S mCherry-actin macrophages in medium containing PI to assess cell death. In this assay, the number of particles internalized by each cell could be determined, and then the subsequent effect of those particles on the cell determined. The data shows that a single particle is sufficient to induce cell death, but only 20% of cells with one particle actually die within 16 hours (Fig. 7A). As the number of particles per cell increased, the frequency of death also increased. This assay also allowed us to assess how long it took cells to die as a function of particle load. The data indicates a direct relationship between particle number and the length of time it takes for cells to die (Fig. 7B). The more particles are internalized, the faster cell death is induced. For example, if a cell internalizes greater than 5 particles, cell death is induced within fifteen hours whereas if a cell internalizes only 1 silica particle, it will take double the length of time to induce death (Fig. 7, n = 37 cells). Cells that acquired 4 or more particles invariably died. Thus, the particle load is a significant factor in both the time and extent of particle toxicity.

### Silica is toxic to macrophage cell lines, but not other cell types

While it is clear that macrophages are susceptible to silica toxicity, it is not clear if this is true of other cell types. To address this question, a variety of non-macrophage cell lines were tested for their ability to take up silica particles and the effects of the particles on the cells. Non-pulmonary, epithelial (HeLa, MDCK, and NIH-3T3) or carcinoma (B16F1 and MtLN3) cell lines (Table 2) showed no cell death after 24 hours of exposure to amorphous or crystalline silica particles. Minimal cell death was induced in MLE-12 cells, which, coincidently, are mouse lung type II epithelial cells. It is possible that these other cell types simply did not take up the particles and that is why no toxicity was observed. Therefore, the uptake of particles by each cell line was quantified using ovalbumin-coated particles and the antibody staining assay. All of the non-macrophage cell lines internalized the amorphous silica particles within 16 hours (Table 2). The interaction of COS7 cells with amorphous silica particles was imaged by time lapse video microscopy and while some COS7 rounded up after particle interaction, no cells were stained with propidium iodide during 16 hours of exposure (Movies S2 and S3). These results indicate that macrophages are especially sensitive to silica particle toxicity. It will be important to ultimately determine what is different about the macrophage that causes death of these otherwise robust cells.

**Table 2:** Many cell lines take up silica particles, but only macrophages show extreme sensitivity.

Cell line	Description	Cell Death (% Membrane Permeability*)			Particle Uptake‡	
		Control (cells alone)	Crystalline silica	Amorphous silica	% Internalized Particles†	Particles/Cell
**MHS** (mouse)	Macrophage	**17**	**100**	**100**	**81**	**4.2**
B16F1 (mouse)	skin melanoma	**0.45**	**0**	**0.15**	**85**	**8**
**Cos7** (monkey)	kidney fibroblast	**0**	**1.3**	**5**	**79**	**6**
**HeLa** (human)	cervical epithelial	**0**	**0**	**0**	**39**	**9**
**MDCK** (dog)	kidney epithelial	**0**	**0**	**0**	**93**	**6.9**
**MTLN3** (mouse)	mammary	**0**	**0**	**0**	**98**	**5.1**
**NIH3T3** (mouse)	embryo fibroblast	**0**	**0**	**0**	**59**	**8.3**

Cells treated with crystalline silica or 3 µm amorphous silica [25 µg/cm^2^].

*Cell death induced at 24 hours, determined by membrane permeability (PI positive).

‡Uptake measurements at 16 hours after silica exposure.

†% internalized equals the number of internalized particles divided by the total number of cell associated particles.

## Discussion

There is extensive evidence that crystalline silica is toxic to both lung tissue and cells in culture. However, the toxicity of amorphous silica is not nearly as well studied. Environmental exposure to amorphous silica is far less common due to its relatively rare presence, so there is almost no epidemiological data in which humans are exposed to amorphous silica that is not contaminated by crystalline silica. There are conflicting published results on the toxicity of amorphous silica exposure to cells and tissues [17], [18]. Instillation of amorphous silica into the lungs of rats had short-term, but less long-term, effects on lung macrophages and lung tissue than crystalline silica [8], [19]. On the other hand, in human lung epithelial cells, amorphous silica was found to cause a greater inflammatory response than crystalline silica [20]. In no case has the actual uptake of amorphous silica actually been examined and correlated with the effects of the particles on cells; the flow cytometry assays used in the past cannot distinguish between surface-bound particles and internalized particles.

Recently, there has been renewed interest in silicosis with work focusing on the issue of the multi-protein complex known as the inflammasome [21]. When resident alveolar macrophages interact with silica, an inflammatory response is elicited and inflammatory cytokines such as interleukin 1-beta and tumor necrosis factor-alpha are released [22]. This induced inflammation can activate NALP3, a member of the cytoplasmic Nod-like receptor family that regulates the activity of Caspase-1 via formation of the inflammsome. Activated Caspase-1 triggers the cleavage of proinflammatory cytokines, interleukin 1-beta and interleukin 18 for subsequent activation and secretion, which is likely to be part of the pathway leading to silicosis [21]. Our work has focused on the proximal events of amorphous silica-cell interaction and the immediate processing of the silica containing phagosome.

In order to assess the effects of amorphous silica on cells in culture, we felt that it was important to determine whether particles were internalized by each cell type. We have recently shown that silica particles are internalized by macrophages and developed an assay to measure the phagocytic process [11]. This assay requires coating of the particles with ovalbumin (non-opsonized) or antibody (opsonized) and then assessing the accessibility of particles to secondary antibodies in fixed cells. Internalized particles cannot bind antibody and thus are not stained, so only external particles become fluorescent. In the course of examining the mechanics of uptake of particles, we discovered two new ways to quantify uptake of particles that are based upon the uniform large size of the particles. First, we examined the involvement of actin polymerization in particle uptake. It is well known that actin polymerization is required for phagosome formation in FcγRIIA receptor-mediated phagocytosis [14]. Macrophage cells expressing mCherry-actin clearly showed an accumulation of actin filaments at the site of cell contact with the particles (Fig. 2). Similar to the opsonized uptake process, these filaments rapidly disassembled as the particle became internalized, leaving a black hole in the cytoplasm where the particle is displacing the cytoplasmic background of non-filamentous mCherry-actin. This allowed the quantification of particle uptake by examining time-lapse video images of the process and quantifying when each bead was taken up. This assay indicated that opsonized, non-opsonized, and naked latex or silica particles were all taken up by actin-mediated phagocytosis, and all were on roughly the same time scale.

We have previously shown that silica causes damage to internal membranes allowing the leakage of endo-lysosomal material into the cytoplasm [10]. This damage is the earliest event known in silica-induced toxicity. We now show that amorphous silica particles cause the same phenomenon to occur. In the course of carrying out this assay, it was noticed that as the particles were being taken up, they became labeled by the fluorescent dextran that had been pre-loaded into endosomes. This observation means that endosomes containing the dextran were fusing with the incoming phagosome during, or shortly after, internalization. The fusion of endomembranes with phagosomes has long been recognized to occur as part of the normal particle uptake process [23]. Therefore, the same process is occurring during the formation of non-opsonized particle phagosomes. Interestingly, the use of FITC-dextran allowed an indirect assessment of the maturation process since the quenching of the fluorescence due to the reduced pH of the compartment was evident. This experiment also led to another assay for silica particle uptake. Counting the fluorescent particles, and comparing this to the total number of particles allowed us to determine the proportion internalized. The uptake of opsonized and non-opsonized amorphous silica showed similar kinetics as determined by the other uptake assays.

The fact that the dextran labeling and actin association assays showed no difference in the rate of uptake of opsonized and non-opsonized particles was surprising. Previous work had shown that non-opsonized particles were taken up at a slower rate [11]. The difference in the results turned out to be a result of temperature. In order to synchronize the particle exposure for the antibody-staining assay, the cells were chilled while particles were centrifuged onto the cells, and then were warmed and analyzed over time. Following this protocol, the cells warm gradually to 37°C, whereas the dextran fusion and mCherry-actin experiments were carried out with cells maintained at 37°C throughout the assay. Thus, there appears to be a temperature-sensitive step in the uptake of non-opsonized particles, while opsonized particle uptake is relatively temperature independent. More investigation will be required to ascertain what aspect of the internalization process proceeds slowly at reduced temperatures.

The extent and kinetics of cell death induced by the various particle types tested was also measured and found to be similar for amorphous and crystalline silica. Since the particles were acquired from commercial sources, the purity of the material is unknown. To verify that the particles needed to be in contact with the cells in order to be toxic, particles were separated from cells by a permeable membrane. The particles were not toxic in this configuration (Figure 1B), thus supporting the idea that uptake is necessary for toxicity. One of the major advantages of the mCherry-actin and dextran labeling assays is that particle uptake can be measured without fixing the cells. Therefore, the time course of events downstream of uptake can be monitored knowing the precise time and number of uptake events in each cell in the field of view. Using the mCherry-actin assay, we found that a single particle is sometimes sufficient to kill a cell. As more particles are internalized, the likelihood of death increases. This is consistent with findings that the toxicity of silica-based materials is proportional to the surface area of particles to which cells are exposed [18], [24].

While the killing of macrophages by silica particles is well established, the effect of silica on other cell types is less clear. It is possible that the inconsistent results are due to some cells types taking up silica particles while others lack the machinery for phagocytosis. With the availability of several uptake assays, we sought to correlate particle uptake with cellular toxicity for a variety of cell types. None of the cell lines tested showed nearly the sensitivity to silica particles that macrophages display. Only the mouse lung epithelial cell line MLE-12 showed any significant toxicity, however all of the cell lines were able to internalize the particles. Therefore, the difference in sensitivity to silica must be related to some physiological function of macrophages that is different from other cell types. It should be noted that the toxicity of silica in macrophages is dramatically reduced by driving uptake of the particles through the FcγRIIA receptor-mediated uptake pathway [11]. Thus it is not just that macrophages are more sensitive to silica, but that macrophage are more sensitive to silica particles taken up by the non-opsonized particle phagocytosis pathway. More will need to be known about the mechanism of uptake, signaling responses, and vesicle trafficking pathway in these other cell types in order to determine why they are resistant to silica toxicity.

## Supporting Information

Figure S1 Cell death in RAW264.7 macrophages exposed to amorphous silica particle. RAW264.7 macrophage cells were exposed to either 3 µm diameter Allsphere silica particles or no particles (cells alone) in media containing 0.2 µg/ml propidium iodide to measure stained nuclei as an indicator of cell death. Amorphous silica particles induced cell death in a manner similar to that seen with MH-S macrophage cells. The assay was performed in triplicate and error bars represent standard error of the mean.
(1.11 MB TIF)

Figure S2 Phagocytosis of amorphous silica particles by RAW264.7 macrophages at room temperature. AW264.7 macrophage cells maintained at room temperature (approximately 21°C) were exposed to either antibody-coated (opsonized) or ovalbumin-coated (non-opsonized) 3 μm diameter Allsphere silica particles. Cells were fixed and stained with fluorescent antibody at increasing time points to determine the rate of particle uptake. Uptake of non-opsonized particles was slower than opsonized particles, but both were taken up by cells. Error bars represent standard deviation of the mean at each time point.
(1.98 MB TIF)

Movie S1 Release of FITC-dextran from endosomes after silica uptake. Cells were preloaded with FITC-dextran for 90 minutes and then amorphous silica particles were added. DIC and FITC widefield images were acquired showing the release of the dextran from punctate endosomal compartments leading to diffuse staining of the cytoplasm.
(0.79 MB MOV)

Movie S2 Amorphous Silica uptake in MH-S cells. MH-S macrophage cells were incubated in CO2-independent medium containing 0.2 μg/ml propidium iodide at 37°C on the stage of a Zeiss Axiovert 200M microscope. DIC images were acquired every minute for 19.5 hours using a 63x oil immersion objective before and after addition of 3 μm Allsphere silica particles (25 μg/cm2). At the end of the movie, about 60% of cells contained propidium iodide stained nuclei.
(7.94 MB MOV)

Movie S3 Amorphous Silica uptake in COS7 cells. COS7 cells were incubated in CO2-independent medium containing 0.2 μg/ml propidium iodide at 37°C on the stage of a Zeiss Axiovert 200M microscope. DIC images were acquired every minute for 16.5 hours using a 63x oil immersion objective before and after addition of 3 µm Allsphere silica particles (25 μg/cm2). At the end of the movie, none of the cell's nuclei were stained with propidium iodide.
(6.03 MB MOV)
