# Supplementary figures and images for: The Phagocytosis and Toxicity of Amorphous Silica
Source: PLoS One. 2011 Feb 2;6(2):e14647. doi: 10.1371/journal.pone.0014647 (PMC3032735; doi:10.1371/journal.pone.0014647)

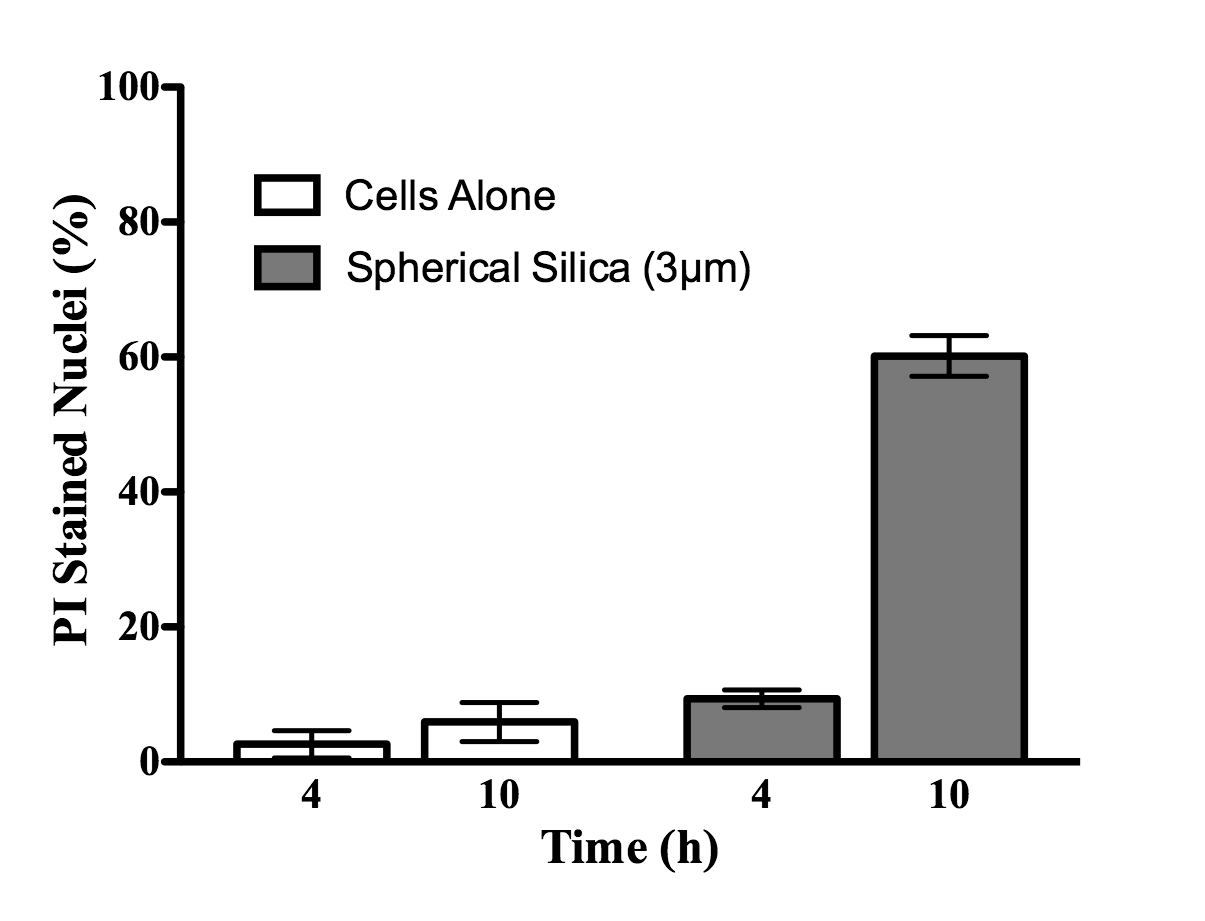

Supplement: Figure S1 — Cell death in RAW264.7 macrophages exposed to amorphous silica particle. RAW264.7 macrophage cells were exposed to either 3 µm diameter Allsphere silica particles or no particles (cells alone) in media containing 0.2 µg/ml propidium iodide to measure stained nuclei as an indicator of cell death. Amorphous silica particles induced cell death in a manner similar to that seen with MH-S macrophage cells. The assay was performed in triplicate and error bars represent standard error of the mean. (1.11 MB TIF) [file pone.0014647.s001.tif]
